# Exposure to Acute Concentration of Malathion Induced Behavioral, Hematological, and Biochemical Toxicities in the Brain of *Labeo rohita*

**DOI:** 10.3390/life15020158

**Published:** 2025-01-23

**Authors:** Sana Ullah, Saeed Ahmad, Muhammad Kashif Ashraf, Muhammad Bilal, Tariq Iqbal, Mahmoud M. Azzam

**Affiliations:** 1 Department of Zoology, Division of Science and Technology, University of Education, Lahore 54000, Pakistan; msf23001074@ue.edu.pk (M.K.A.); msf23001071@ue.edu.pk (M.B.); 2 Department of Zoology, University of Malakand, Dir Lower, Khyber Pakhtunkhwa, Chakdara 18800, Pakistan; abutalhauom@yahoo.com; 3 College of Animal Sciences, Zhejiang University, Hangzhou 310058, China; 0622744@zju.edu.cn; 4 Animal Production Department, College of Food and Agriculture Sciences, King Saud University, Riyadh 11451, Saudi Arabia; mazzam@ksu.edu.sa

**Keywords:** Malathion, rohu, neurotoxicity, behavioral inconsistencies, hematoxicity, biochemical disruption

## Abstract

A surge has been observed in the use of pesticides to boost agricultural yield in order to feed the continuously increasing human population. Different types and classes of broad-spectrum insecticides are in use, and the number is constantly increasing with the introduction of new ones. Keeping in view the broad-spectrum effects of organophosphate pesticides including Malathion (MLN), their use is continuously increasing without appraising their toxic impacts on non-target organisms. The continuous rise in the use of MLN has led to its presence, persistence, and transport to water bodies globally, subsequently affecting commercially valuable aquatic organisms. The current study was conducted to assess MLN-induced hematological and biochemical toxicities in the brain of a commercially valuable indigenous major carp, rohu, *Labeo rohita*. The fish was exposed to an acute concentration of commercial-grade MLN. The LC_50_ of MLN (5 µg/L) led to behavioral inconsistencies and subtle impacts on the hematology (an increase in white blood cells and a reduction in red blood cells, hemoglobin, packed cell volume level, and mean corpuscular hemoglobin concentration) and biochemistry (an increase in reactive oxygen species, lipid peroxidation, activities of antioxidant enzymes (catalase, peroxidase, superoxide dismutase, glutathione, glutathione reductase, glutathione peroxidase, and glutathione-S-transferase) but a reduction in total protein content and activity of Na^+^/K^+^ ATPases) in the brain tissues. MLN also inhibited the activity of Acetylcholinesterase, while it led to an increase in Acetylcholine. Significant changes were observed in the serum biochemical profile; for example, glucose, cholesterol, potassium, urea, and total bilirubin increased, whereas total protein, sodium, chloride, albumin, and inorganic phosphate decreased after exposure. The current study clearly classified MLN as highly toxic to rohu. Therefore, the extra-judicious use of MLN should be strictly supervised. Studies concerning the real-world concentration of pesticides should be carried out on regular basis to mitigate the echoing issue of pesticide-based pollution.

## 1. Introduction

Pesticides are employed to increase agricultural yield and control vector-borne diseases. Approximately 4 million tons of pesticides are employed annually [1]. The enormous expansion in the chemical industry has led to the introduction of hundreds of different pesticides, belonging to different groups and classes. Due to the continuous rise in pesticide use, around two-thirds of global agricultural land is polluted with more than one pesticide, whereas the remaining third is at a high risk [2]. Despite approximately 740,000 cases of human acute poisoning reported in 2020 [3], a significant increase in pesticide use is projected in future [4].

Organophosphate (OPP) is a highly potent class of pesticides, and therefore, it is widely employed. OPPs make up almost 50% of the total insecticides used globally [5]. However, despite this huge amount of employed OPPs, there is no proper disposal management for them, specifically in under-developed or developing countries. In these countries, the OPPs find their way into the air, soil, water, and biospheres, where they are deposited or accumulated. Therefore, OPPs are reported from air, soil, water, and biological components [6,7]. The issue of grave concern is their dumping in aquatic bodies and subsequent accumulation in non-target organisms, including fish. OPPs not only affect the health of the exposed aquatic organisms but also enter the food chain, ultimately affecting their consumers [8].

Malathion (MLN) is an organophosphate. It is known to cause genotoxicity in humans and induces several toxic impacts on aquatic organisms, including hepatic [9], neuronal [10], metabolic [11], and reproductive/developmental disruption [12], but it is still widely utilized. Because of the widespread use of MLN, it is reported from different water bodies across the globe, including India, Spain, Iran, and the Amazonian region [13,14,15,16]. However, there is no study concerning MLN monitoring in aquatic bodies from Pakistan.

Their relatively lengthy lifespan and movement make fish effective bioindicators of long-term and short-term effects of pollutants in various habitats [4]. The sensitive nature of fish makes them a robust animal model for studying behavioral, hematological, physiological, and biochemical impacts of various chemicals [9]. Fish also provide a precise and rapid insight into accumulation of different toxicants, chemicals, or pollutants; their distribution into different tissues; their mechanism of action; and their metabolism [17]. Moreover, fish can reveal adverse outcomes at the individual as well as at the population level; therefore, fish are replacing other animal models for toxicological research. Currently, different fish species are employed to study ecotoxicological outcomes of chemicals on account of their easy maintenance in a laboratory, swift development, and small size; however, readily available and commercially valuable fish species are prioritized on account of their availability and consumption rate [18].

*Labeo rohita* (Hamilton, 1822) is a highly prized game fish due to its high market demand, locally known as rohu. It is one of the most widely consumed fish species in Pakistan and India on account of its taste. It is widely distributed in freshwaters across different Asian countries, including Pakistan, India, Bangladesh, Nepal, Sri Lanka, and Myanmar. Rohu is an herbivorous bottom feeder; however, their fry consume zooplankton. Rohu is found in temperate and tropical regions and breeds around June–July in running water. Having 52 diploid chromosomes, their fecundity varies from 226,000 to 2,794,000, depending on the weight and length of their ovaries. Rohu possess an ossified endoskeleton and are widely utilized as an animal model due to their higher consumption rate and easy availability. Tanika [19] provided a detailed description of rohu.

MLN-induced toxicities in different fish species are well studied, including *Channa punctatus*, *Clarias batrachus*, *Carassius auratus*, *Oreochromis mossambicus*, *Oncorhynchus mykiss*, *Colossoma macropomum*, *Ictalurus furcatus*, *Danio rerio*, and *Solea senegalensis* [5,20,21,22,23,24,25,26,27]. However, their toxic effects on the brain of commercially valuable fish species, such as rohu (*Labeo rohita*), are still unknown. Therefore, the current study was aimed to appraise the behavioral, hematological, and biochemical effects of MLN on the brain of rohu. The current study will fill the gap to improve the ecotoxicological assessment of MLN. By involving multiple biomarkers, the current study will enhance our understanding of subtle effects of MLN from various standpoints. Furthermore, this study will widen toxicity markers employed for characterizing oxidative stress development, which subsequently leads to different toxic effects in the brain of rohu, a widely employed model organism.

## 2. Materials and Methods

### 2.1. Fish Handling, Acclimatization, and Water Quality

A total of 250 fingerlings (weight: 6.9 ± 1.32 g; length: 8.8 ± 1.21 cm) of rohu were transported through closed-system live hauling to the laboratory from a local hatchery. The fish were carefully conditioned to avoid stress or any damage and then distributed in aquaria (60 cm × 30 cm × 30 cm). Before starting the experiment, the fish were acclimatized for 15 days. The fish were fed a basal protein diet (35%-protein-based food in small dried pelleted form at the rate of 5% of their body weight) twice a day.

The model species were kept in ground water of ambient quality. Different water quality parameters including temperature, hardness, ammonia, pH, dissolved oxygen (DO), and conductivity were assessed on a daily basis through a water quality meter (Horiba U10), pH meter, and DO meter. The water quality parameters were within permissible ranges: pH ranged from 6.7 to 7.5, DO ranged from 6.2 to 7.5 mg/L, temperature ranged between 24.4 and 25.9 °C, ammonia was observed to be <0.25ppm, total hardness ranged between 162 and 179 mg/L, and conductivity ranged from 240 to 290 µs/cm.

### 2.2. Test Chemical and LC_50_ Determination

Commercial-grade MLN (for further detail, see Table S1) was purchased from the local market because it is the most widely used grade of MLN [28]. Acetone was used for preparing a stock solution of MLN. The required dilution amount was then used in the study. The control group received 0.005% acetone of the same volume used for experimental group, with no toxic effects. The LC_50_ of MLN was determined in a semi-static method via probit analysis.

For determining LC_50_, fish were divided into 7 aquaria (10 fish/aquarium). The fish in these 9 aquaria were exposed to MLN at different concentrations, namely 0 µg/L, 1 µg/L, 2 µg/L, 3 µg/L, 4 µg/L, 5 µg/L, 6 µg/L, 7 µg/L, and 8 µg/L. No mortality was observed at 0 µg/L, 1 µg/L, and 2 µg/L. Mortality occurred after exposure to 3 µg/L and higher concentrations. The data regarding mortalities were noted in these aquaria for 96 h. The 5 µg/L concentration of MLN was observed to be LC_50_, causing 50% mortality of rohu after 96 h, as shown in Table S2 and Figure S1 [9].

### 2.3. Experimental Design

The experiment was conducted in a semi-static closed system (12 h–12 h light–dark cycle). A total of 150 active, healthy, and uniform-sized fish (irrespective of gender) were divided into 2 groups (75 fish each for the control and experimental groups). The fish were stocked in both control and treated (experimental/exposed) groups. The experiment was conducted in triplicate. The fish in the control were not exposed to MLN, whereas the fish in experimental group were exposed to LC_50_ (5 µg/L) of MLN.

The water was changed on a daily basis before restoring the acute concentration of MLN. Fish were collected from both the control and treated groups after 24, 48, 72, and 96 h (*n* = 3 per aquarium, N = 9 per group). The collected fish were anesthetized using MS222 (60 mg/L). The blood was collected using syringes through caudal vein puncture, and the brain tissues were removed on an icebox. The tissues were stored at −20 °C for further analysis.

### 2.4. Behavioral Analysis

The fish in both the control and experimental groups were keenly observed for any behavioral alterations and inconsistencies such as hyperactiveness, hypoactiveness, aggregation, equilibrium loss, darting movement, adapting vertical position, aggression, etc., by following well-documented research [29,30].

### 2.5. Hemato-Biochemical Analysis

The blood was collected through caudal vein puncture by using sterile needles and syringes (25 G—0.5 mm (needle diameter or thickness) × ½ inch or 13 mm (needle length)). The collected blood was transferred to EDTA tubes to prevent coagulation. The blood was checked for parasitic infestation under a microscope to ensure infection-free blood for analysis. EDTA (1.26 mg/0.6 mL) was used as an anticoagulant. Because of the small size of the fish, blood from three fish specimens was pooled together for further analysis. The hematological parameters including white blood cells (WBCs), red blood cells (RBCs), hemoglobin (Hb), packed cell volume (PCV), mean corpuscular volume (MCV), mean corpuscular hemoglobin (MCH), and mean corpuscular hemoglobin concentration (MCHC) were assayed by following David et al. [31]. The procured blood was kept on ice for an hour and then centrifuged for 10 min at 3000 rpm for serum isolation. The isolated serum was stored at −80 °C for analyzing different biochemical parameters including glucose, total protein, cholesterol, inorganic phosphate, chloride, urea, bilirubin, sodium, chloride, potassium, and albumin by following Qadir et al. [32].

### 2.6. Biochemical Analysis

On account of the small size of the brain, three samples were pooled together to acquire the required weight for biochemical analysis from the same aquarium. A total of 3 specimens were collected from an aquarium, and 9 specimens were collected from the same group (*n* = 3, N = 9) after 24, 48, 72, and 96 h. ROS and LPO were assayed by following Ullah et al. [9], AChE was evaluated by following Bibi et al. [33], total protein was appraised by following Lowry et al. [34], and antioxidant enzymes were assayed using standard protocols such as catalase and peroxidase by following Chance and Maehly [35], superoxide dismutase by following Kakkar et al. [36], glutathione reductase by following Carlberg and Mannerik [37], glutathione peroxidase by following Mohandas et al. [38], glutathione-s-transferase by following Habig et al. [39], and glutathione content by following Jollow et al. [40]. A complete and detailed description of these protocols is provided in Table S3. Na^+^/K^+^ ATPases were assayed by following Mukerjee et al. [41].

### 2.7. Statistical Analysis

The data (expressed as mean ± S.E.) obtained were analyzed using MS Excel (V. 2016) and Statistix (V. 8.1). To test the homogeneity of variance (multiple variance analysis), the data were analyzed using one-way analysis of variance (ANOVA) followed by least-significant difference (LSD). A *p* value less than 0.5 was considered statistically significant.

## 3. Results

### 3.1. Effect of Malathion on the Behavior of Rohu

Exposure to Malathion led to different behavioral inconsistencies in rohu (as shown in Table 1). After an hour, the fish started jumping and showed hyper-activeness in the exposed group. After some hours of exposure, equilibrium loss was observed with erratic swimming. After 24 h, the exposed fish were observed coming to the surface for breathing and air gulping. During the later stages of the experiment, the fish started aggregating at the bottom corner of the aquaria, adapting vertical position, and cessation of feeding in the experimental group. The fish collected for biochemical and hematological analysis were observed to have external hemorrhage, color change, and secreted mucus. However, no behavioral disruption was observed in the control group.

### 3.2. Effects of Malathion Exposure on the Brain of Rohu

**Figure 1:**
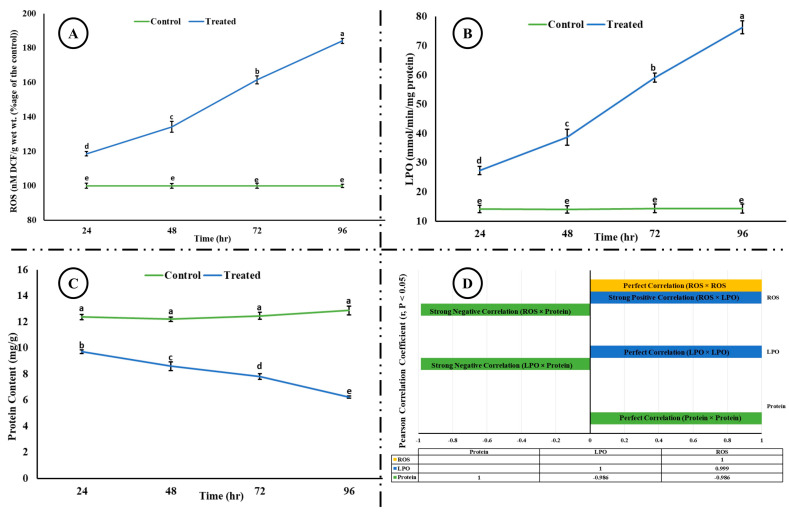
(**A**) ROS, (**B**) LPO, (**C**) total protein content, and (**D**) correlation among ROS, LPO, and total protein content in the brain of rohu at different time intervals. The different superscripted letters in (**A**), (**B**), and (**C**) indicate a significant difference at *p* < 0.05 (ANOVA followed by LSD test). (**D**) shows Pearson correlation (considering r values > 0.5 as significant at *p* < 0.05) between ROS, LPO, and total protein content. A strong negative correlation (−0.986) was observed between ROS and total protein, and LPO and total protein, whereas a strong positive correlation (0.999) was observed between ROS and LPO.

Exposure of rohu to the acute concentration of Malathion led to a significant increase (*p* < 0.05) in the level of reactive oxygen species and lipid peroxidation; however, a significant reduction in the level of total protein content was observed in the brain of rohu (Figure 1). A strong positive correlation (*r* > 0.5) was observed between ROS and LPO; however, both ROS and LPO showed a strong negative correlation (*r* > −0.5) with total protein content.

**Figure 2:**
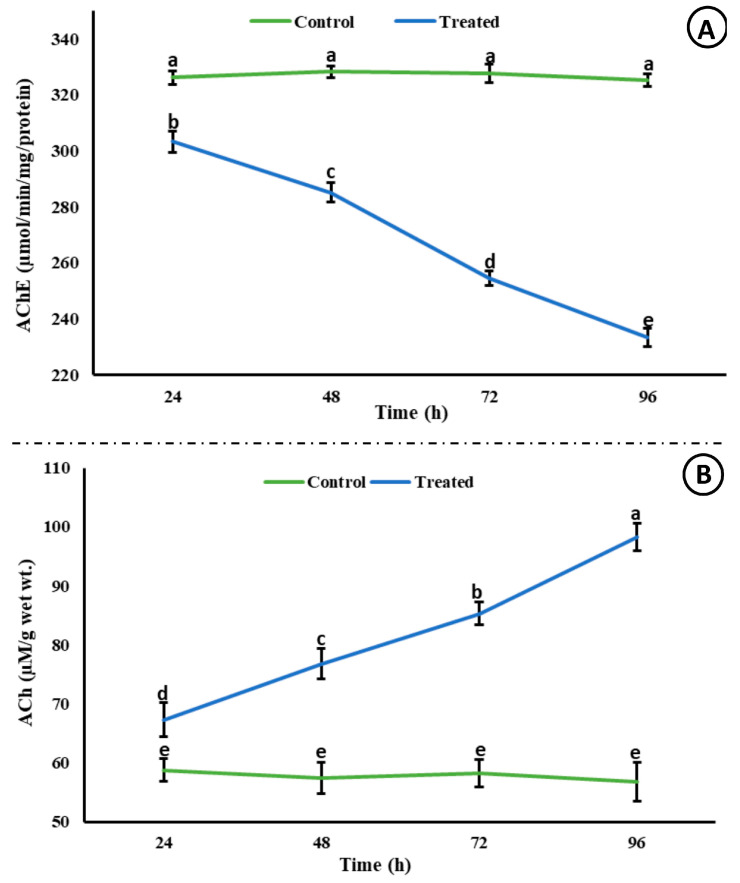
Acetylcholinesterase activity (µmol/min/mg/protein) and ACh (µM/g wet wt.) in the brain of rohu at different time intervals. The data are presented as the mean ± SE and were analyzed using ANOVA followed by LSD. The readings with different superscripted letters are significantly different at *p* < 0.05.

**Figure 3:**
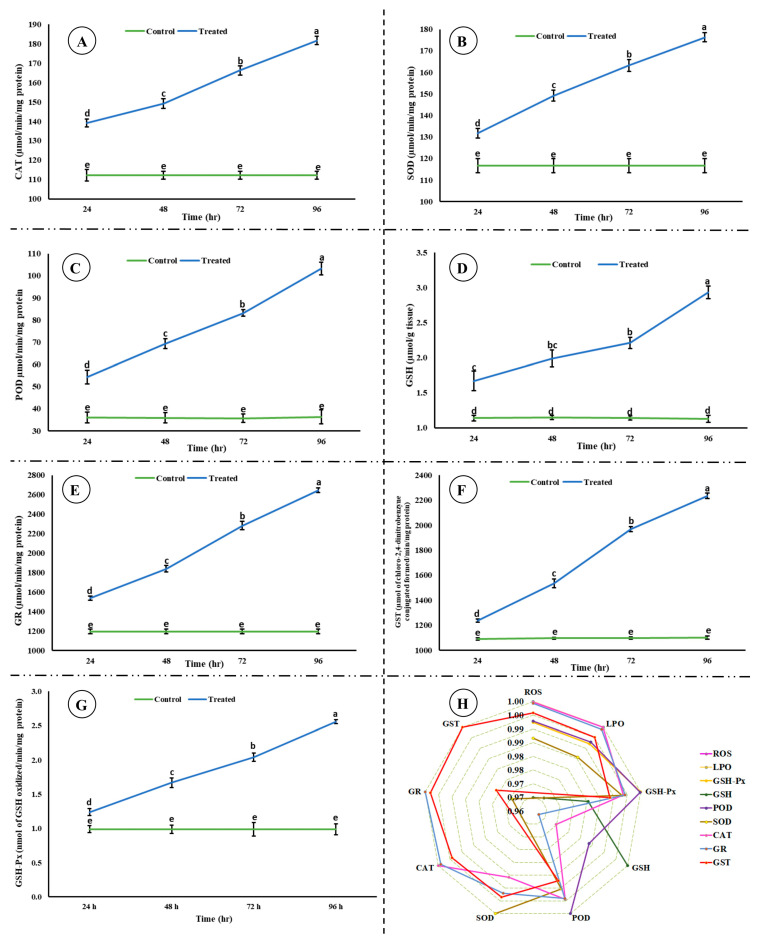
Activities of antioxidant enzymes ((**A**) catalase, (**B**) superoxide dismutase, (**C**) peroxidase, (**D**) glutathione, (**E**) glutathione reductase, (**F**) glutathione-S-transferase, (**G**) glutathione peroxidase, and (**H**) correlation among the studied antioxidant enzymes, ROS, and LPO) in the brain of rohu at different time intervals. (**A**–**G**) The data are presented as the mean ± SE and were analyzed using ANOVA followed by LSD. The readings with different superscripted letters are significantly different at *p* < 0.05.

**Figure 4:**
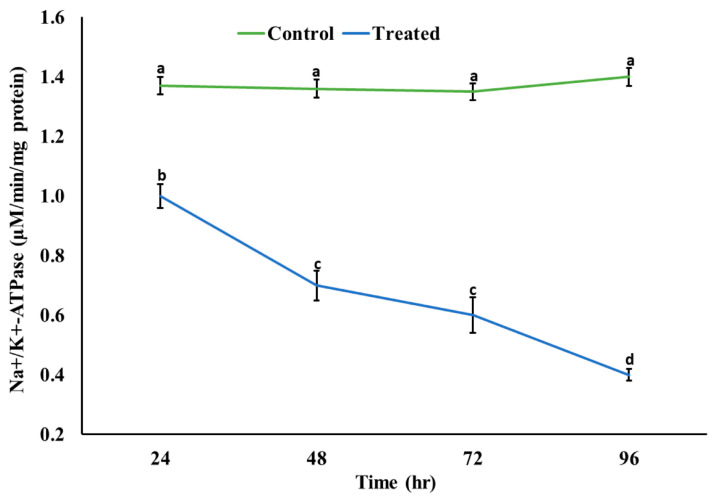
Activity of sodium/potassium ATPases in the brain of rohu at different time intervals. The data are presented as the mean ± SE and were analyzed using ANOVA followed by LSD. The readings with different superscripted letters are significantly different at *p* < 0.05.

A significant decrease (*p* < 0.05) in the activity of AChE was observed; however, a significant increase (*p* < 0.05) in the level of ACh was observed in the brain of rohu after exposure to an acute concentration of Malathion (Figure 2). A significant increase (*p* < 0.05) was observed in the activities of antioxidant enzymes (Figure 3) in the exposed group compared to the control group, whereas a significant decrease (*p* < 0.05) was observed in the activity of Na^+^/K^+^ ATPases (Figure 4).

### 3.3. Effects of Malathion Exposure on the Hematology and Blood Biochemistry

**Figure 5:**
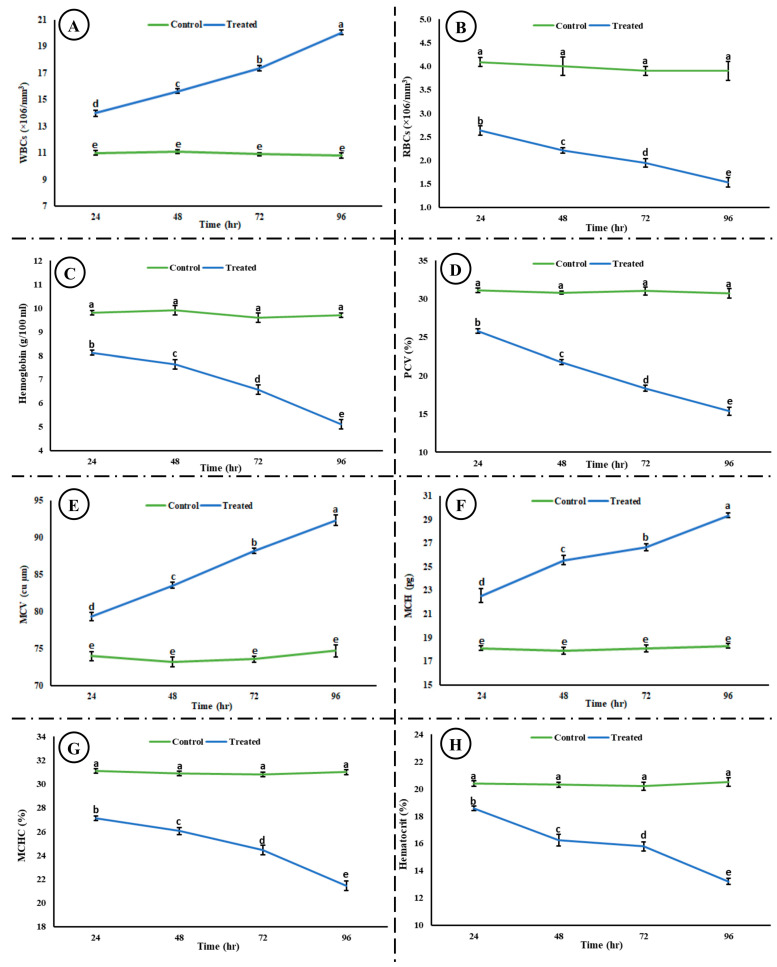
Hematological ((**A**) WBCs, (**B**) RBCs, (**C**) hemoglobin, (**D**) PCV, (**E**) MCV, (**F**) MCH, (**G**) MCHC, and (**H**) hematocrit) toxicity induced in rohu after exposure to an acute concentration of Malathion. The data are presented as the mean ± SE and were analyzed using ANOVA followed by LSD. The readings with different superscripted letters are significantly different at *p* < 0.05.

**Figure 6:**
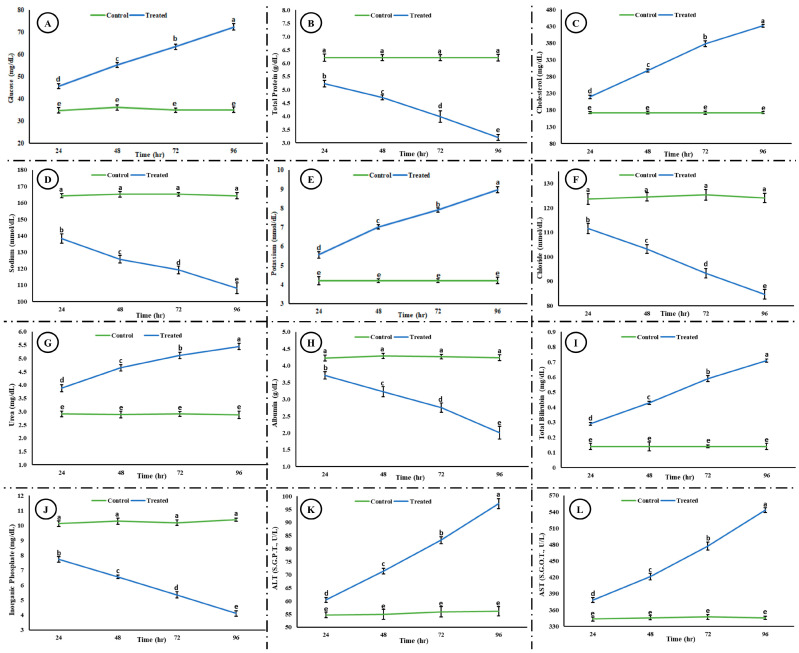
Blood biochemical profile ((**A**) glucose, (**B**) total protein, (**C**) cholesterol, (**D**) sodium, (**E**) potassium, (**F**) chloride, (**G**) urea, (**H**) albumin, (**I**) total bilirubin, (**J**) inorganic phosphate, (**K**) ALT, and (**L**) AST) after exposure to an acute concentration of Malathion. The data are presented as the mean ± SE and were analyzed using ANOVA followed by LSD. The readings with different superscripted letters are significantly different at *p* < 0.05.

Exposure to the acute concentration of Malathion induced different toxic effects on the hematological and blood biochemical parameters in the exposed group. Figure 5 shows the induced hematological changes in the control and treated groups after 24, 48, 72, and 96 h of exposure to an acute concentration of Malathion. A significant time-dependent increase (*p* < 0.05) was observed in white blood cells (WBCs), mean corpuscular hemoglobin (MCH), and packed cell volume (PCV) in the exposed group after exposure to MLN. However, a significant decrease (*p* < 0.05) was observed in red blood cells (RBCs), hemoglobin (Hb), packed cell volume (PCV), mean corpuscular hemoglobin concentration (MCHC), and hematocrit (Hct) in the experimental group after exposure to MLN. No change was observed in the control group after 24, 48, 72, and 96 h. Figure 6 shows the blood biochemical profile in both the control and experimental groups. A significant increase was observed in the glucose, cholesterol, potassium, urea, total bilirubin, ALT, and AST, whereas a significant decrease was observed in the total protein, sodium, chloride, albumin, and inorganic phosphate in the exposed group.

## 4. Discussion

In light of the current study, MLN can be classified as highly neurotoxic to rohu. The neurotoxic effect was obvious from the inhibition of Acetylcholinesterase (AChE) and the accumulation of Acetylcholine (ACh) in the brain of rohu. Moreover, the behavior of the exposed fish was altered, disrupted, inconsistent, and unusual compared to fish from the control group, which behaved naturally, and their movement was well coordinated. The fish in the control group were observed to be vigilant and alert even to the slightest disturbance, whereas the exposed fish showed inactiveness. The exposed fish exhibited darting, erratic, and irregular swimming. They lost their equilibrium, which might be associated with the failure of the sensory system and disrupted vision and olfaction. The disrupted swimming behavior might be associated with the inhibition of AChE. The inhibition of AChE led to the accumulation of ACh, leading to hyperstimulation. MLN showed neurotoxic effects on rohu, disrupting their neurotransmitter function and brain development as observed in previous studies [42]. The neurotoxic effects led to different alterations in the behavior of rohu, which is well aligned with previous studies [43]. Cessation of feeding might be linked with the disruption of digestion and ammonia accumulation [44]. Fasciculation, muscle weakness and cramps, and flaccidity might lead to erratic and disturbed swimming. The dead fish showed an excessive release of mucus, external hemorrhage, and color change. Mucus secretion acts as a primary line of defense by preventing toxicants, pathogens, and harmful substances from entering the fish’s body [45]. Moreover, mucus contains immunoglobulin, antimicrobial peptides, and enzymes; therefore, mucus neutralizes toxicants and helps in their removal [46]. Similarly, mucus might be linked with detoxification, trapping, and removing MLN as an adaptation to protect the skin and heal wounds as a protective covering [47]. The fish from the experimental groups secreted mucus, and its secretion increased in a time-dependent manner, which is in correspondence with external hemorrhage observed.

Reactive oxygen species are reactive molecules, containing oxygen, produced during cellular processes, and they play key roles in homeostasis and cell signaling. However, when above permissible levels, they lead to oxidative stress and consequently lead to cellular and histological damage. They also attack macromolecules such as nucleic acid, oxidants, lipids, and proteins, and subsequently lead to cytotoxicity and disruption of physiological and biochemical processes [9]. An increase was observed in ROS, which was in correspondence with the increased lipid peroxidation (strong and positively correlated, r = 0.999). LPO is a vital indicator of oxidative damage and is employed to appraise oxidative stress. The current study of increase in ROS and LPO is congruent with previous studies [9,48].

A significant increase was observed in antioxidant enzymes activities, which might be a protective response of rohu against MLN. Antioxidant enzymes maintain cellular homeostasis and act as a primary line of defense. The increase in the activities of antioxidant enzymes is congruent with the increase in ROS, as antioxidant enzymes neutralize ROS and prevent oxidative damage. Antioxidant enzymes mitigate oxidative stress; for example, SOD catalyzes superoxide radicals into oxygen and H_2_O_2_, CAT converts H_2_O_2_ into molecular oxygen and water, POD neutralize H_2_O_2_ and lipid peroxides, GSH-Px defends tissues against H_2_O_2_ or ROOH and is associated with epithelium renovation, GSH modulates stress-induced LPO by working as a reducing substrate, GR again converts oxidized GSH to a reduced state, and GST catalyzes xenobiotic conjugates to tri-peptide GSH [9]. The current study reporting an increase in the activities of antioxidant enzymes is well aligned with previous studies [48]. The increase in the activities of GR and GSH are well coordinated and congruent with each other.

A significant decrease in protein contents might be associated with oxidative damage, which directly damages amino acid residues and subsequently damages protein. It might be embodied to the capacity of MLN to alter the protein structure. The decrease might also be associated with the inhibition of protein synthesis, disruption of translation, or apoptosis, leading to protein breakdown as a cellular degradation process. It might also be associated with fulfilling energy requirements under stress. The result is in congruence with previous studies [44]. Similarly, a significant decrease was observed in the activity of Na^+^/K^+^ ATPases, which might be associated with the disturbed Na^+^-K^+^ pump on account of pH-induced inhibition [41]. This subsequently leads to enzymatic dysfunction and neurotoxicity, as shown by previous studies [41,49].

Leukocytosis might be associated with an activated immune system to fight stress, tissue damage, and inflammation; to facilitate repair processes; and as a defense against secondary pathogens or damage [50]. Increase in the WBCs is reported by previous studies in response to different pesticides [30,51]. The decrease in RBCs might be associated with hemolysis, inhibition of erythropoiesis, elevated eryptosis, or disruption of osmoregulation [50]. Decreased hemoglobin might be associated with impaired production of hemoglobin, inhibition of hemoglobin-synthesizing enzymes, oxygen deprivation, oxidative stress, or as a secondary effect of anemia [39]. A decrease in MCHC and PCV might be linked with hemodilution, anemia, or impaired erythropoiesis. The increase in MCV and MCH (accompanied with decreased MCHC, hemoglobin, and RBCs) indicated hypochromic anemia due to inhibited erythropoiesis and impaired oxygen carrying capacity [52]. The same disturbances in hematological parameters are well reported by previous studies [51,53].

MLN exposure led to a disturbed serum biochemical profile. A decrease in sodium and an increase in potassium might be linked with disruption of ion balance, disturbed osmoregulation, renal dysfunction, or metabolic failure. A decrease in serum protein might be associated with protein catabolism, impaired nutrient intake, decreased protein synthesis, or protein degradation after exposure to MLN. An increase in urea corresponded to decreased total protein, which might be associated with protein catabolism. An increase in serum cholesterol might be attributed to cortisol or adrenaline release in response to MLN exposure, as indicated in previous studies [30]. Decreased inorganic phosphate might be attributed to kidney dysfunction, disrupted metabolic pathways, phosphate transporters alteration, and energy imbalance. Elevated total bilirubin was congruent with a decrease in red blood cells, as an increase in total bilirubin level indicates increased breakdown of RBCs or disrupted liver function. Disrupted liver function is also indicated by elevated AST and ALT in a time-dependent manner. Previous studies have also reported disrupted serum biochemistry after exposure of fish to pesticides [54].

## 5. Conclusions

The current study clearly classified MLN as a potent neurotoxic and hematotoxic pesticide. Therefore, the injudicious and indiscriminate use of MLN should be strictly monitored, avoided, and prohibited if required to ensure a thriving population of commercially valuable fish species. Moreover, the biomarkers including behavior, biochemistry (ROS, LPO, total protein content, and antioxidant enzymes), and hematology can be employed as potent ecotoxicological endpoints for chemical risk assessment and biomarkers for bio-monitoring of aquatic organisms, whereas AChE and ACh can be considered as potential neurotoxicological endpoints. The current study was limited to behavioral, hematological, and biochemical aspects. Therefore, further research on cholinergic receptors, monoamine activities, and molecular cross-talk is suggested to fully elucidate MLN’s mode of action and provide ample information to devise preventive strategies for reducing the potential hazardous impacts of MLN.

## Figures and Tables

**Table 1:** Behavioral inconsistencies in *Labeo rohita* after exposure to Malathion.

Behavioral Inconsistencies	Groups	
	Control	Treated
Jumping	Absent	Present
Erratic swimming	Absent	Present
Hyperactiveness	Absent	Present
Equilibrium loss	Absent	Present
Darting movement	Assent	Present
Air gulping	Absent	Present
Coming to the water surface to breathe	Absent	Present
Aggregation at the bottom corner of aquaria	Absent	Present
Hyperventilation	Absent	Present
Hypoactiveness	Absent	Present
Motionless state	Absent	Present
Adapting vertical position	Absent	Present
Cessation of feeding	Absent	Present
Secretion of mucus	Absent	Present
Hemorrhage (external)	Absent	Present
Color change	Absent	Present

## Data Availability

All data associated with this study are provided in this article and its Supplementary Materials.

## Supplementary Materials

The following supporting information can be downloaded at: https://www.mdpi.com/article/10.3390/life15020158/s1. Table S1: Chemical composition of the Malathion (50% EC—emulsifiable concentrate) by weight (CAS R.No. 121-71-5). Table S2: Determination of lethal concentration (LC_50_) of Malathion against *L. rohita* for 96 h. Table S3: A complete and detailed description of these protocols for biochemical parameters. Figure S1: Toxicity evaluation of Malathion against rohu, *L. rohita* ((A) log concentration × percent mortality; (B) log concentration × probit mortality).
