# Supplementary material for: Exposure to Acute Concentration of Malathion Induced Behavioral, Hematological, and Biochemical Toxicities in the Brain of Labeo rohita
Source: Life (Basel). 2025 Jan 23;15(2):158. doi: 10.3390/life15020158 (PMC11856734; doi:10.3390/life15020158)
Supplement: Supplementary file 1 [file life-15-00158-s001.zip › life-3382028-supplementary.pdf]

Table S1. Chemical composition of the Malathion (50% EC – Emulsifiable Concentrate) by weight (CAS R.No. 121-71-5)

S. No.	Ingredients	Components	% W/W
1	Active	Malathion: 0,0-dimethyl phosphorodithioate of diethyl mercaptosuccinate	50.00
2	Inert	Xylene range aromatic solvent	50.00
		Total	100.00

Table S2. Determination of lethal concentration (LC_{50}) of Malathion against *L. rohita* for 96 h

S. No	Conc. (µg/L)	Log Conc.	Fish exposed	Mortality (%)	Probit's Mortality Value (95% CI)
1	2.0	0.477	10	0	0.00
2	3.0	0.544	10	10	3.72
3	4.0	0.602	10	30	4.48
4	5.0	0.653	10	50	5.00
5	6.0	0.699	10	70	5.52
6	7.0	0.740	10	90	6.28
7	8.0	0.778	10	100	8.09

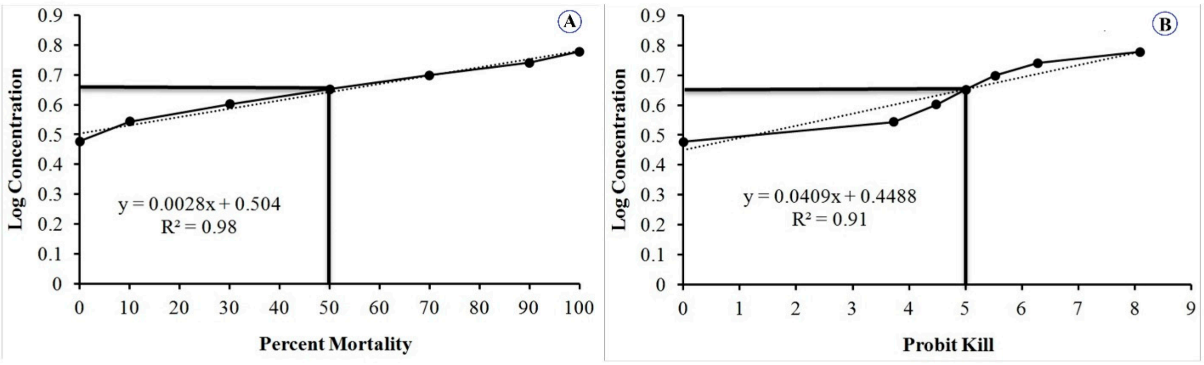

Fig. S1. Toxicity evaluation of Malathion against rohu, *L. rohita* [(A) Log concentration × Percent mortality; (B) Log concentration × Probit mortality].

Table S3. A complete and detailed description of these protocols for biochemical parameters

S. No.	Biochemical parameters	Detailed protocols
1	Reactive Oxygen Species (ROS)	Level of the reactive oxygen species (ROS) was assessed through DCF-DA (2',7'-dichlorofluorescein diacetate). The pooled samples of the brain (0.1-1 g wet wt.) were incubated in 10 μM DCF-DA (100 ml) in a water bath (37 °C) for 30 min in methanol. The DCF fluorescence was observed using spectrofluorometer at 488 nm and 525 nm excitation and emission wavelengths, respectively. The standard curve (0–500 nM DCF) was used to obtain fluorescence values.
2	Lipid Peroxidation (LPO, μmol/ min/ mg protein)	For preparing reaction mixture (1 ml), 0.58 ml phosphate buffer (pH 7.4, 0.1 M), 0.2 ml ascorbic acid (100 mM), and 0.02 ml ferric chloride (100 mM) were mixed with 0.2 ml supernatant. The prepared solution was incubated at 37 °C in a water bath for an hour, and 10% trichloroacetic acid (1 ml) was added to stop the reaction. All the tubes were then boiled for 20 min in a water bath after addition of thiobarbituric acid (1 ml), then cooled via ice bath, and were then centrifuged for 10 min at 2500×g. The change in absorbance of the solution was recorded (535 nm) after one min using a spectrophotometer.
3	Total Protein Content	A total of 90 mg brain was taken out, homogenized (phosphate buffer), and were centrifuged for 20 min at 10,000 rpm at 4 °C. The stock solution (1 mg/ml) of the standard BSA (bovine serum albumin) was prepared by dissolving BSA (15 mg) in distilled water (15 ml). A solution (No. 1) was prepared by mixing 2% Na2CO3 (50 ml) and 0.1 N NaOH (50 ml) solution. Another solution (No. 2) was prepared by mixing 1.56% CuSO4 (10 ml) solution with 2.37% Sodium Potassium Tartrate (10 ml) solution. Folin-Ciocalteau reagent was prepared by mixing 2 ml commercial reagent with water (equal amount). An alkaline solution was procured after mixing Solution No. 2 (2 ml) with Solution No. 1 (100 ml). After that, the sample was thawed, and 0.1 ml sample was mixed with 1 ml alkaline solution, incubated (10 min),

		then 1:1 Folin-Ciocalteau phenol reagent was added into each tube, vortexed for proper mixing, and after 30 min incubation, the optical density was observed (595 nm) through spectrophotometer. The concentration of protein was calculated using BSA standard curve.
4	Catalase (CAT, μmol/ min/ mg protein) and Peroxidase (POD, μmol/ min/ mg protein)	For evaluating the CAT activity (briefly), 50 mM phosphate buffer (2.5 ml, pH 5), and 5.9 mM H2O2 (0.4 ml) were mixed with 0.1 ml enzyme extract. For POD activity evaluation (briefly), 50 mM phosphate buffer (2.5 ml, pH 5), 40 mM H2O2 (0.3 ml), and 20 mM guaiacol (0.1 ml) were mixed with 0.1 ml enzyme extract for making reaction solution. The change in the absorbance of the reaction solution was recorded after one min using spectrophotometer (CAT at 240 nm, and POD at 470 nm)
5	Superoxide Dismutase (SOD, μmol/ min/ mg protein)	The reaction mixture was prepared by mixing 0.1 ml phenazine methosulphate (186 μM), 1.2 ml sodium pyrophosphate buffer (0.052 mM, pH 7), and supernatant (0.3 m). NADH (0.2 ml, 780 μM) was added to initiate enzyme reaction and was stopped by addition of glacial acetic acid (1 ml) after one min. Chromogen amount formed was measured via color intensity recording using spectrophotometer (560 nm).
6	Glutathione Reductase (GR, μmol NADPH oxidized/ min/ mg protein)	Reaction solution was prepared by mixing 0.1 M phosphate buffer (1.65 ml, pH 7.6), 0.5 mM EDTA (0.1 ml), 0.1 mM NADPH (0.1 ml), 1 mM oxidized glutathione (0.05 ml), and supernatant (0.1 ml). The absorbance change was recorded after one min using spectrophotometer (340 nm).
7	Glutathione Peroxidase (GSH-Px, nmol of GSH oxidized/ min/ mg protein)	The reaction solution was procured after mixing 0.1 M phosphate buffer (1.49 ml, pH 7.4), 1 mM sodium azide (0.1 ml), glutathione reductase (1 IU/ml, 0.05 ml), 1 mM GSH (0.05 ml), 1 mM EDTA (0.1 ml), 0.2 mM NADPH (0.1 ml), 0.25 mM H2O2 (0.01 ml), and 10% homogenate (0.1 ml) in a total volume of 2.0 ml. NADPH disappearance was recorded at 25 °C (340 nm).
8	Glutathione-s-transferase (GST, μmol of chloro-2,4-dinitrobenzyne	The reaction solution was prepared by mixing 0.1 M phosphate buffer (1.475 ml, pH 6.5), 1 mM 2,4-Dinitrochlorobenzene (CDNB, 0.025 ml), 1 mM reduced glutathione (0.2 ml), and 10% homogenate (0.3 ml) in a

	conjugated formed/ min/ mg protein)	total volume of 2.0 ml. The change in the absorbance was recorded (340 nm).
9	Glutathione (GSH, μmol/ g tissue)	The mixture was prepared by mixing 0.1 M phosphate buffer (1.475 ml, pH 6.5), 1 mM CDNB (0.025 ml), 1 mM reduced glutathione (0.2 ml), and 10% homogenate (3 ml) in a total volume 2.0 ml. The change in absorbance was recorded (340 nm).
